# HLA profiles and susceptibility to Alveolar echinococcosis: evidence from a large cohort with population and household controls

**DOI:** 10.1080/22221751.2026.2744492

**Published:** 2026-10-15

**Authors:** Lynn Peters, Zuotang Ye, Alexander Grunenberg, Giuseppe Zenatti, Wanjie Jiang, Yasemin Özkan, Nele Wellinghausen, Teresa Rangel-Vivar, Benedikt Haggenmüller, Andreas Binzberger, Wolfgang Kratzer, Marko Kornmann, Erik Rasbach, Daniel Fürst, Beate Grüner

**Affiliations:** a Department of Internal Medicine III, Division of Infectious Diseases, University Hospital Ulm, Ulm, Germany; b Department of Infectious Disease Immunology and Allergology, MVZ Labor Ravensburg GbR Labor Dr. Gärtner, Ravensburg, Germany; c Department of Radiology, University Hospital Ulm, Ulm, Germany; d Department of Internal Medicine I, University Hospital Ulm, Ulm, Germany; e Department of General and Visceral Surgery, University Hospital Ulm, Ulm, Germany; f Institute of Clinical Transfusion Medicine and Immunogenetics Ulm, German Red Cross Blood Transfusion Service, Baden Wuerttemberg – Hessen, Germany; g University Hospital Ulm, Ulm, Germany; h Institute of Transfusion Medicine, University of Ulm, Ulm, Germany

**Keywords:** Alveolar echinococcosis, echinococcus multilocularis, HLA, MHC, genetic susceptibility

## Abstract

Alveolar echinococcosis (AE) is an emerging and potentially fatal helminthic disease. Despite widespread environmental contamination with infective *Echinococcus multilocularis* (EM) eggs in endemic areas, human AE remains rare, suggesting an important role of host genetic susceptibility. In this case–control study, HLA variation was assessed by amplicon-based next-generation sequencing in 360 patients with AE and compared with (i) 1,440 population controls matched, where feasible, on age, sex, and place of residence, and (ii) 100 unrelated household controls to account for shared environmental exposure to EM. Analyses included age- and sex-adjusted Firth-penalized logistic regression, linkage disequilibrium (LD)-aware haplotype analyses, simulation-based maximum-score tests, and Benjamini–Hochberg false discovery rate correction. At two-field resolution, C*07:01 was associated with increased AE odds (OR 2.10; adjusted *p* = 0.006), whereas B*18:01 was associated with reduced odds (OR 0.49; adjusted *p* = 0.014). Haplotype analyses identified an extended LD-defined background involving A*29, C*16, or both, frequently combined with B*44, DRB1*07, or DQB1*02. A*29 carriage corresponded to the A01–A24 supertype category. A*29–DQB1*02 remained significant after within-model and study-wide correction (OR 3.92; adjusted *p* = 0.005 and 0.039, respectively), while C*16–B*44–DQB1*02 remained significant after within-model correction (OR 2.83; adjusted *p* = 0.041) but not after study-wide correction. Exploratory analyses of early-onset AE (≤35 years; n = 63) suggested associations with A*31:01 (OR 2.86) and DQB1*05:03 (OR 3.44), although neither remained significant after multiple-testing correction. These findings support a contribution of individual HLA alleles and extended haplotypic backgrounds to AE susceptibility within a multifactorial host–environment model.

## MANUSCRIPT

## Background

Alveolar echinococcosis (AE) is a chronic, potentially fatal helminthic disease caused by the cestode *Echinococcus multilocularis* (EM) and is endemic in parts of the northern hemisphere, including southern Germany. The adult cestode resides in the intestines of red foxes (*Vulpes vulpes*) and sheds infective eggs with the faeces. Following accidental ingestion, the larval stage (metacestode) can establish in the human liver and grow invasively in a tumour-like manner [1].

Molecular epidemiological studies have demonstrated considerable contamination of kitchen gardens and food products with EM DNA in endemic areas,[2,3] yet human AE remains rare (0.1–2.8 cases per 100,000 inhabitants) [4–8]. This discrepancy suggests that, beyond exposure, host factors – including genetic susceptibility – contribute to infection and disease development.

The human leucocyte antigen (HLA) system is central to adaptive immunity: HLA class I molecules present endogenous peptides to CD8+ T cells, whereas HLA class II molecules present exogenous peptides to CD4+ T cells. Given its extensive polymorphism, HLA variation influences antigen presentation and thereby shapes inter-individual differences in immune responses, including susceptibility or resistance to infection. Moreover, strong linkage disequilibrium (LD) between HLA loci can result in associations involving inherited allele combinations rather than individual alleles alone.

Previous studies investigating HLA associations with AE in Switzerland, France, Germany, China, Turkey, Russia, Alaska, and Latvia, [9–16] have yielded heterogeneous and sometimes conflicting results. Interpretation is limited by small sample sizes, differences in typing resolution and regional HLA backgrounds, incomplete locus coverage, limited consideration of haplotypic context, and the absence of exposure-matched control groups.

The primary objective of this study was to assess associations between HLA allele groups, alleles, and haplotypes and susceptibility or resistance to AE in a large German cohort. Patients were compared with population-based and unrelated household controls, analysed jointly and separately; household controls were included to better account for shared environmental exposure. HLA homozygosity, class I supertypes, and early-onset disease were examined in complementary exploratory analyses. As AE is diagnosed at a mean age of approximately 54 years in Europe, [17–20] early-onset disease was defined as diagnosis at ≤35 years. Because later disease manifestation may be influenced by immunosenescence, comorbidities, or immunosuppressive treatment, earlier onset may represent a phenotype in which host genetic susceptibility plays a more prominent role.

Beyond association testing, robust HLA signals may offer insight into immunological mechanisms relevant to AE. If replicated, HLA markers could serve as one component in combined host–exposure risk models and thereby inform more targeted surveillance approaches, with potential long-term relevance for precision public health.

## Methods

### Study design and participant recruitment

This observational case–control study was performed at the University Hospital Ulm, Germany. Adult patients (≥18 years) with probable or confirmed AE according to the WHO-IWGE case definition [21] who attended the AE outpatient clinic between March 2024 and May 2025 were invited to participate. After written informed consent, EDTA whole blood was collected for genetic analyses. HLA profiles of patients were compared with two control groups: (i) population-based controls from a registry of healthy volunteer stem cell donors and (ii) unrelated household controls to approximate shared environmental exposure.

The study was approved by the Ethics Committee of the Ulm University (No. 109/20 and amendment No. 20/24).

### Population-based controls

Population-based controls were derived from an existing registry of HLA profiles from healthy volunteer stem cell donors. For each AE patient, four controls were selected and matched by sex, age, and region based on the patient’s recorded place of residence 10 years prior to diagnosis, to approximate the likely region of infection. Due to the age restrictions for stem cell donor registration, exact age matching was not feasible and registry controls were slightly younger. AE was not specifically excluded in registry controls, as it is not part of routine donor assessment; however, given the low prevalence of AE even in endemic regions, the probability of including undiagnosed cases was considered negligible.

### Household controls: eligibility and screening

To include a control group with comparable environmental exposure, patients were invited to enrol an unrelated household member, typically a partner. Household controls were eligible if they were ≥18 years, not biologically related to the patient, and had shared the same household with the patient for ≥5 years prior to the patient’s AE diagnosis, reflecting the incubation period of AE. After written informed consent, AE was excluded by two serological tests (Echinococcus IgG ELISA (ESR107G), Virion/Serion, Würzburg, Germany; E. multilocularis Em2-Em18 IgG ELISA, Bordier Affinity Products SA, Crissier, Switzerland) and abdominal ultrasound performed by examiners experienced in AE imaging. EDTA whole blood was collected for genetic analyses.

### Outcomes

The primary endpoint was the association of HLA alleles and haplotypes with AE case status in comparisons of patients versus population-based controls and versus unrelated household controls. Additional analyses included an HLA supertype analysis and an early-onset subgroup analysis (≤35 years at first diagnosis).

### HLA typing and data processing

Genomic DNA was isolated from EDTA whole blood using magnetic bead-based extraction on the chemagic platform (PerkinElmer chemagen, Baesweiler, Germany). Immunogenetic diagnostics were performed using an IVD-CE marked, amplicon-based next-generation sequencing assay on an Illumina platform (MiSeq; Illumina, San Diego, CA, USA). Briefly, target loci were amplified by PCR, purified using MagSi-NGSprep Plus beads (Magtivio, Nuth, Netherlands), and underwent a second PCR to add molecular barcodes and adapters prior to pooling. Sequencing was performed using MiSeq v3 chemistry (2 × 300 bp). Sequence analysis and HLA allele assignment were carried out using Sequence Pilot SEQNEXT-HLA (JSI, Ettenheim, Germany) and in-house software (EVP, IKT-Ulm, Germany). Typing results were reviewed for quality and consistency.

### Statistical analysis

Analyses were performed using R version 4.5.2 (R Foundation for Statistical Computing, Vienna, Austria), including the haplo.stats package version 1.9.8.2 and the logistf package version 1.26.1.

For the primary overall-control comparison, population and household controls were pooled without weighting, with each individual contributing equally; additional analyses were performed separately for each control group.

Continuous and categorical variables were summarized using standard descriptive statistics and compared using parametric, non-parametric, chi-square, or Fisher’s exact tests, as appropriate. Regression models were adjusted for age per 10-year increase and sex. Firth-penalised logistic regression was used for allele, supertype, and early-onset models to reduce small-sample bias and provide more stable estimates in the presence of sparse data or separation. Results are reported as adjusted odds ratios (ORs) with 95% confidence intervals (CIs). Multicollinearity was assessed using variance inflation factors (VIF) and conditional models where appropriate. To account for multiple testing, the Benjamini–Hochberg false discovery rate (BH-FDR) procedure was applied to limit the expected proportion of false-positive findings among statistically significant results. Tests were two-sided; raw *p* < 0.05 indicated nominal association and BH-FDR-adjusted *p* < 0.05 significance after multiple-testing correction.

One- and two-field HLA carrier frequencies were compared between AE patients and each control group, with BH-FDR correction applied separately by resolution and comparison. HLA allele groups and alleles with an overall carrier frequency >1% were included. Nominal signals from the overall-control comparison were jointly modelled at each resolution; population-control models used the same predictors, whereas additional household-specific models were exploratory.

For selected HLA markers, post hoc power for the household-control comparison was estimated using exact two-sided Fisher tests, observed household carrier frequencies, and effect estimates from the population-control comparison.

Two-field HLA homozygosity was examined using the number of homozygous loci, the presence of any homozygous locus, and locus-specific homozygosity, with BH-FDR correction applied to the five locus-specific tests.

Because HLA loci are in strong linkage disequilibrium (LD) – i.e. certain alleles are inherited together more often than expected by chance – haplotype-based analyses were additionally performed. Pairwise and three-locus haplotypes were estimated using the expectation–maximisation algorithm (haplo.em) implemented in haplo.stats. Haplotypes with an overall estimated frequency <1% were excluded from individual analyses. Additive haplotype regression (haplo.glm) estimated effects per additional haplotype copy relative to the most frequent haplotype within each locus combination. Regression *p*-values were adjusted within each combination and across the respective pairwise or three-locus family. Reference-independent, age- and sex-adjusted haplotype-specific score tests (haplo.score) provided pointwise simulated *p*-values, while maximum-score simulation *p*-values were BH-FDR-corrected separately across the ten pairwise and ten three-locus combinations.

Two exploratory analyses were performed. First, HLA class I supertypes were examined by assigning two-field HLA-A and HLA-B alleles to the 12 predefined categories described by Sidney et al., [22] which group alleles with shared or overlapping peptide-binding specificities. Participants were classified as carriers if at least one allele was assigned to the respective category. All 12 supertype carrier variables were jointly evaluated in an age- and sex-adjusted Firth model, with BH-FDR correction across the 12 supertype coefficients.

Second, early-onset AE was investigated as a phenotype in which host genetic susceptibility might play a more prominent role. HLA markers identified in the preceding early-onset screening were evaluated in separate one- and two-field models. BH-FDR correction was applied both across all screened markers at the respective resolution and within each selected multivariable model. Robustness was assessed using leave-one-case-out analyses and conditional refitting of correlated predictors.

## Results

### Participant characteristics

We compared 360 AE patients with 1,540 controls, including 100 unrelated household controls and 1,440 population controls. Mean age was 63.8 years in patients (SD 16.3; median 66.0; IQR 22.75), 64.6 years in household controls (SD 13.7; median 67.0; IQR 17.0), and 59.1 years in population controls (SD 11.9; median 65.0; IQR 14.0). Age did not differ significantly between patients and household controls, whereas population controls were significantly younger than both patients (*p* < 0.001) and household controls (*p* < 0.001). Sex distribution did not differ across groups (*p* = 0.276): 58% of patients were female, compared with 50% of household controls and 58% of population controls.

### Allele groups and alleles

#### Univariate analyses

One-field HLA allele groups and two-field alleles were compared between 360 patients with AE and 1,540 overall controls. Separate comparisons with population and household controls are provided in Supplementary Table S1.

Several nominal class I associations were observed (Table 1). C*07:01 showed the lowest raw *p* value and was more frequent in AE patients than in overall controls (35.8% vs 26.8%; OR 1.52, 95% CI 1.20–1.94; raw *p* < 0.001). Additional nominal risk associations involved A*29/A*29:02, B*08/B*08:01, B*38/B*38:01, and C*16/C*16:01, whereas B*18/B*18:01 showed nominal protective associations.

**Table 1.** Significant raw results of the univariate analysis. Markers with central two-sided Fisher or Pearson p raw < 0.05 for AE versus overall controls are shown including respective allele group. For each control comparison, BH-FDR correction was applied separately to the complete set of one-field and two-field tests. Frequencies are carrier prevalences (n/N), ORs are unadjusted odds ratios with 95% confidence intervals. Abbreviations: AE, alveolar echinococcosis; BH, Benjamini-Hochberg; CI, confidence interval; FDR, false discovery rate; OR, odds ratio (protective factor = green, risk factor = red).

HLA marker	AE patients, n/N (%)	Overall controls, n/N (%)	OR (95% CI)	*p* raw	*p* FDR (BH)
A*29	30/360 (8.3%)	74/1540 (4.8%)	1.801 (1.159–2.798)	**0**.**008**	0.211
A*29:02	30/360 (8.3%)	65/1540 (4.2%)	2.063 (1.317–3.231)	**0**.**001**	0.132
A*66	0/360 (0.0%)	18/1540 (1.2%)	0.000 (0.000–0.967)	**0**.**045**	0.439
A*66:01	0/360 (0.0%)	18/1540 (1.2%)	0.000 (0.000–0.967)	**0**.**045**	0.868
B*08	89/360 (24.7%)	289/1540 (18.8%)	1.422 (1.084–1.865)	**0**.**011**	0.211
B*08:01	88/360 (24.4%)	289/1540 (18.8%)	1.400 (1.067–1.839)	**0**.**015**	0.625
B*18	25/360 (6.9%)	160/1540 (10.4%)	0.644 (0.415–0.998)	**0**.**047**	0.439
B*18:01	24/360 (6.7%)	158/1540 (10.3%)	0.625 (0.400–0.976)	**0**.**037**	0.856
B*38	24/360 (6.7%)	63/1540 (4.1%)	1.675 (1.031–2.719)	**0**.**035**	0.439
B*38:01	24/360 (6.7%)	63/1540 (4.1%)	1.675 (1.031–2.719)	**0**.**035**	0.856
C*07	199/360 (55.3%)	775/1540 (50.3%)	1.220 (0.969–1.536)	0.091	0.557
C*07:01	129/360 (35.8%)	413/1540 (26.8%)	1.524 (1.195–1.943)	**<0**.**001**	0.132
C*16	35/360 (9.7%)	89/1540 (5.8%)	1.756 (1.166–2.643)	**0**.**006**	0.211
C*16:01	29/360 (8.1%)	65/1540 (4.2%)	1.988 (1.263–3.129)	**0**.**003**	0.175
DRB1*01	47/360 (13.1%)	267/1540 (17.3%)	0.716 (0.513–0.9996)	**0**.**049**	0.439
DRB1*01:01	36/360 (10.0%)	224/1540 (14.5%)	0.653 (0.450–0.947)	**0**.**024**	0.762
DRB1*14	28/360 (7.8%)	69/1540 (4.5%)	1.798 (1.141–2.834)	**0**.**010**	0.211
DRB1*14:01	16/360 (4.4%)	31/1540 (2.0%)	2.264 (1.225–4.186)	**0**.**007**	0.390
DQB1*05	99/360 (27.5%)	428/1540 (27.8%)	0.985 (0.762–1.274)	0.911	1.000
DQB1*05:01	51/360 (14.2%)	287/1540 (18.6%)	0.721 (0.522–0.995)	**0**.**046**	0.868
DQB1*05:03	27/360 (7.5%)	71/1540 (4.6%)	1.678 (1.060–2.654)	**0**.**026**	0.762

Among class II markers, several nominal associations were observed: DRB1*14/DRB1*14:01 and DQB1*05:03 were more frequent in AE patients, whereas DRB1*01/DRB1*01:01 and DQB1*05:01 were less frequent.

In the exploratory household-control comparison, DRB1*01:01 was less frequent in AE patients than in household controls and showed a nominal protective association (10.0% vs 19.0%; OR 0.47, 95% CI 0.26–0.87; raw *p* = 0.014). The unadjusted protective estimate was more pronounced than in the separate population-control comparison (OR 0.67), and DRB1*01:01 was the only marker showing raw *p* < 0.05 in both separate control comparisons. A*29:02 (OR 2.90, 95% CI 0.88–9.84; raw *p* = 0.067) and C*16 (OR 2.59, 95% CI 0.90–7.46; raw *p* = 0.069) showed risk estimates in the same direction as in the population-control comparison and narrowly missed nominal significance. None of the remaining markers nominally associated in the overall-control comparison reached nominal significance against household controls. Notably, C*07:01 – the strongest overall-control signal – had a similar carrier prevalence in AE patients and household controls.

Post hoc power analyses indicated that the household-control comparison had only 10.6–43.7% power to detect the effect sizes observed for selected HLA markers in the population-control analysis. At 80% power, the minimum detectable risk ORs were approximately 2.00 for B*08, 2.88 for B*38, and 3.65 for C*16. Therefore, nonsignificant household-control findings should be interpreted as inconclusive rather than as evidence against associations observed in the larger control comparisons.

None of the observed associations remained significant after BH-FDR correction (adjusted *p* ≥ 0.132).

#### Multivariate analyses

The primary models comparing AE patients with overall controls are presented in Tables 2 and 3; corresponding analyses using population and household controls are provided in Supplementary Tables S2a-2f.

**Table 2.** AE versus overall controls: one-field allele-group model (protective factor = green, risk factor = red).

Allele group	OR	95% CI	*p* raw	*p* FDR (BH)	VIF
A*29	1.34	0.75-2.33	0.319	0.319	1.45
A*66	0.11	<0.01-0.82	**0**.**026**	0.069	1.01
B*08	1.47	1.11-1.94	**0**.**008**	0.064	1.03
B*18	0.70	0.44-1.07	0.098	0.131	1.02
B*38	1.69	1.01-2.75	**0**.**045**	0.090	1.01
C*16	1.65	0.96-2.76	0.068	0.109	1.46
DRB1*01	0.81	0.57-1.13	0.218	0.250	1.03
DRB1*14	1.77	1.10-2.80	**0**.**020**	0.069	1.01

Logistic regression was adjusted for age (per 10-year increase) and sex.

Abbreviations: FDR, false discovery rate; BH, Benjamini-Hochberg; CI, confidence interval; OR, odds ratio.

**Table 3.** AE versus overall controls: two-field allele model (protective factor = green, risk factor = red).

Allele	OR	95% CI	*p* raw	*p* FDR (BH)	VIF
A*29:02	1.54	0.79-2.94	0.197	0.338	1.84
A*66:01	0.11	<0.01-0.81	**0**.**024**	0.090	1.01
B*08:01	0.75	0.48-1.19	0.223	0.338	2.62
B*18:01	0.49	0.29-0.78	**0**.**003**	**0**.**014**	1.17
B*38:01	1.70	1.01-2.76	**0**.**045**	0.124	1.01
C*07:01	2.10	1.39-3.17	**<0**.**001**	**0**.**006**	2.69
C*16:01	1.61	0.82-3.08	0.165	0.338	1.84
DQB1*05:01	1.03	0.57-1.79	0.912	0.912	3.59
DQB1*05:03	1.31	0.66-2.44	0.430	0.473	1.75
DRB1*01:01	0.72	0.38-1.42	0.340	0.416	3.58
DRB1*14:01	1.67	0.70-4.01	0.246	0.338	1.75

Logistic regression was adjusted for age (per 10-year increase) and sex.

Abbreviations: FDR, false discovery rate; BH, Benjamini-Hochberg; CI, confidence interval; OR, odds ratio.

In the one-field model, carriage of B*08 (OR 1.47, 95% CI 1.11–1.94; raw *p* = 0.008), B*38 (OR 1.69, 95% CI 1.01–2.75; raw *p* = 0.045), and DRB1*14 (OR 1.77, 95% CI 1.10–2.80; raw *p* = 0.020) was nominally associated with increased odds of AE. A*66 was nominally associated with lower odds of AE (OR 0.11, 95% CI <0.01–0.82; raw *p* = 0.026). However, A*66 was not observed among AE patients, making this estimate highly dependent on sparse data and requiring cautious interpretation. None of the one-field associations remained statistically significant after within-model BH adjustment, although the adjusted *p* values for B*08, A*66, DRB1*14, and B*38 ranged from 0.064 to 0.090.

In the two-field model, B*18:01 was associated with approximately 50% lower odds of AE (OR 0.49, 95% CI 0.29–0.78; raw *p* = 0.003; BH-adjusted *p* = 0.014), whereas C*07:01 was associated with approximately twofold higher odds of AE (OR 2.10, 95% CI 1.39–3.17; raw *p* < 0.001; BH-adjusted *p* = 0.006). A*66:01 (OR 0.11, 95% CI <0.01–0.81; raw *p* = 0.024) and B*38:01 (OR 1.70, 95% CI 1.01–2.76; raw *p* = 0.045) showed the same direction as their corresponding one-field allele group respectively; however, neither association remained significant after BH adjustment (adjusted *p* = 0.090 and 0.124, respectively).

No severe multicollinearity was detected in the primary models, with all VIFs below 5. Nevertheless, moderate dependence was observed between the linked B*08:01 and C*07:01 signals. In conditional analyses, B*08:01 was associated with higher AE odds after exclusion of C*07:01 (OR 1.44, 95% CI 1.08–1.91; *p* = 0.012), while C*07:01 remained associated with AE after exclusion of B*08:01 (OR 1.71, 95% CI 1.32–2.21; *p* < 0.001). These conditional analyses indicate a shared LD-defined signal; independent causal effects of B*08:01 and C*07:01 cannot be disentangled in this dataset.

Analyses restricted to population controls yielded closely similar effect estimates, as expected because population controls constituted the majority of the combined control group. In these models, the associations of B*18:01 and C*07:01 remained significant after within-model BH adjustment, and B*08 additionally met the adjusted significance threshold in the one-field model. Full results are reported in the Supplementary Material.

When the predictor sets from the primary analyses were applied to the household-control comparison, none of the examined HLA markers was significantly associated with AE. In additional household-specific exploratory models based on the corresponding univariate findings, C*17 was nominally associated with lower AE odds in the one-field analysis (OR 0.15, 95% CI 0.03–0.69; raw *p* = 0.015), as was A*02:01 in the two-field analysis (OR 0.55, 95% CI 0.35–0.88; raw *p* = 0.012). Neither association remained significant after BH adjustment (adjusted *p* = 0.076 and 0.073, respectively).

#### Homozygosity

At two-field resolution, overall HLA homozygosity was not associated with AE: patients and controls had a similar mean number of homozygous loci (0.44 versus 0.47; adjusted OR per additional homozygous locus 0.95, 95% CI 0.83–1.09; *p* = 0.476). Results were likewise null in separate comparisons with population and household controls and in sensitivity analyses evaluating any or locus-specific homozygosity; no locus-specific association remained significant after BH-FDR correction (Supplementary Table S3a and 3b).

### Haplotype analysis

Among the pairwise haplotype analyses, 24 haplotypes showed nominal associations in the age- and sex-adjusted regression models (Supplementary Table S4a). A*29–DQB1*02 showed the strongest and most robust association, with an estimated frequency of 3.13% in AE patients versus 1.00% in controls and an adjusted OR of 3.92 (95% CI 1.93–7.98; raw *p* < 0.001). This was the only pairwise haplotype that remained significant after both within-model and study-wide regression-based BH-FDR correction (BH-FDR-adjusted *p* = 0.005 and 0.039, respectively).

The maximum-score analyses identified a broader, internally consistent pattern. Five of the ten locus-pair maximum-score tests remained significant after BH-FDR correction across the ten pairwise models. The strongest positive scores in these models were observed for A*29–B*44, A*29–C*16, A*29–DRB1*07, A*29–DQB1*02, and C*16–DRB1*07. These haplotypes had pointwise simulated *p*-values of 0.0002–0.0011, while the corresponding model-level maximum-score *p*-values ranged from 0.0129 to 0.0227 and were BH-FDR-adjusted to 0.0454 for each model. The regression estimates for A*29–C*16, A*29–DRB1*07, and C*16–DRB1*07 showed concordant nominal risk associations, with ORs ranging from 1.98 to 2.46, whereas A*29–B*44 did not reach nominal regression significance (*p* = 0.067) despite being the strongest score haplotype within the A–B model.

Related combinations involving C*16–B*44, C*16–DQB1*02, and B*44–DQB1*02 also showed nominally increased ORs and pointwise simulated score *p*-values <0.05. However, none remained significant after regression-based BH-FDR correction, and none was supported by the corresponding locus-pair maximum-score test. Other nominal findings included C*07–B*08 and DRB1*14–DQB1*05 haplotypes, which contained components corresponding to risk signals observed in the multivariable allele analyses. Neither regression association survived BH-FDR correction, and neither corresponding locus-pair maximum-score test was significant.

Among the three-locus analyses, 14 haplotypes showed nominal associations in the age- and sex-adjusted regression models (Supplementary Table S4b). Only C*16–B*44–DQB1*02 remained significant after within-model BH-FDR correction (3.31% in AE patients versus 1.36% in controls; OR 2.83, 95% CI 1.48–5.41; raw *p* = 0.0016; within-model BH-FDR-adjusted *p* = 0.041), whereas no individual haplotype coefficient survived the study-wide BH correction. The recurring risk pattern involved an extended LD-defined haplotype background containing A*29, C*16, or both, in varying combinations with B*44, DRB1*07, and DQB1*02. Seven related haplotypes showed nominally increased ORs ranging from 2.29 to 2.83. In the complementary score analyses, haplotypes from this extended background yielded the strongest positive score in nine of the ten locus-combination models: three contained both A*29 and C*16, three contained A*29 without C*16, and three contained C*16 without A*29. Their pointwise simulated score *p*-values were ≤0.0023. All nine corresponding locus-combination models also remained significant in the maximum-score analysis after BH-FDR correction across the ten three-locus models (adjusted *p* = 0.0015–0.0495). Interestingly, DRB1*07 showed a context-dependent pattern: while it occurred in risk-associated haplotypes containing A*29 or C*16, it showed nominally protective regression estimates in combination with B*57.

Selected pairwise and three-locus haplotypes from the regression and score-based analyses are summarized in Figure 1. The visualization combines haplotype composition, estimated frequencies in AE patients and overall controls, and adjusted effect estimates, thereby highlighting the recurring A*29/C*16-associated risk background. Complete results are provided in Supplementary Tables S4a and S4b.

**Figure 1.**
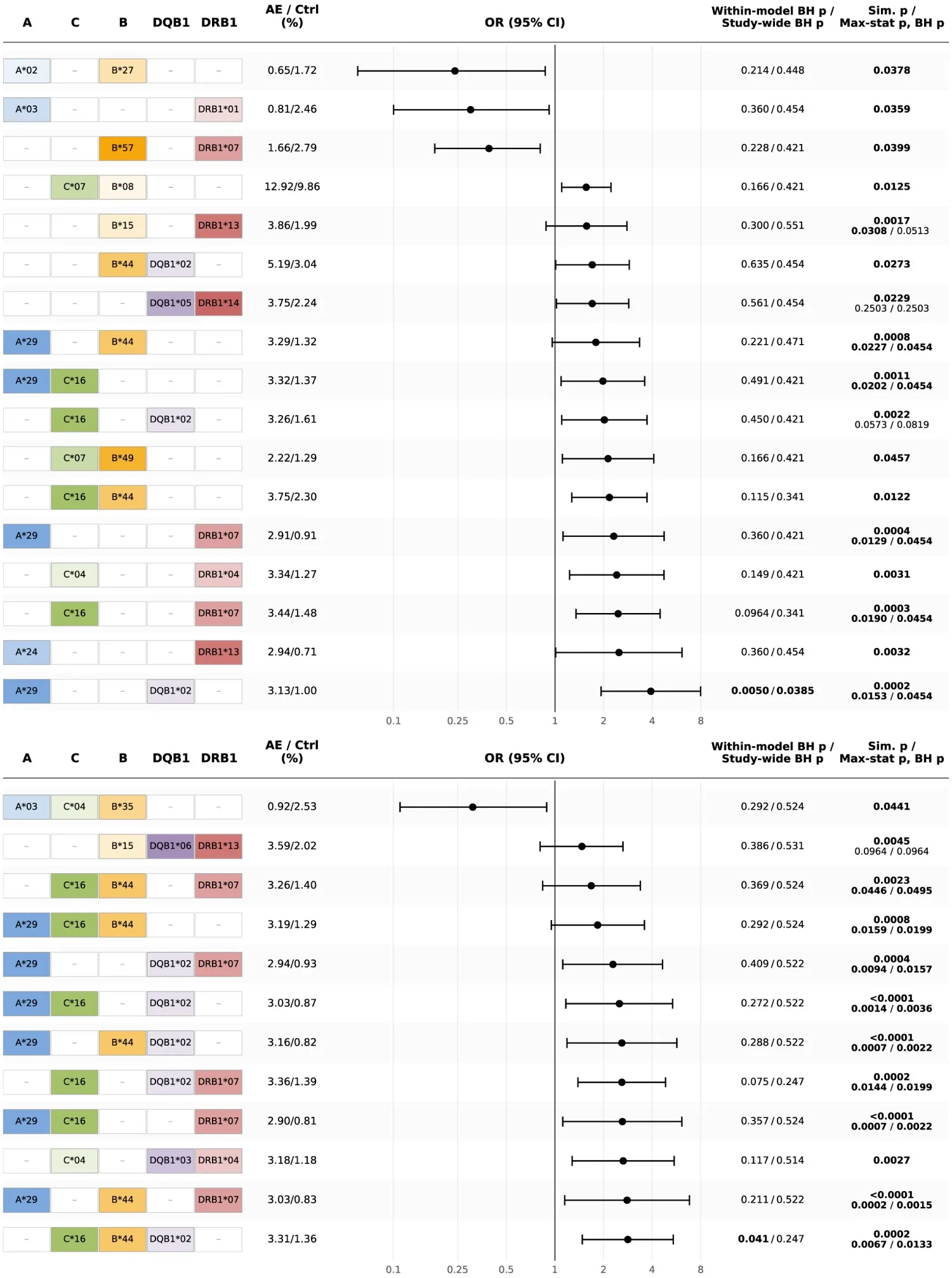
Composition and effect estimates of HLA haplotypes associated with alveolar echinococcosis.

Selected two-locus (Figure 1(a)) and selected three-locus haplotypes (Figure 1(b)) illustrating the recurring risk background and representative protective associations; complete results are provided in Supplementary Tables S4a and S4b. Colored boxes indicate the allele groups included in each haplotype, with locus-specific color families and allele-specific shades. AE/Ctrl denotes haplotype frequencies in AE patients and overall controls, estimated using the expectation–maximisation algorithm. Adjusted ORs and 95% CIs were derived from additive haplotype-regression models adjusted for age and sex and are relative to the locus-combination-specific reference haplotype. ORs are plotted on a logarithmic scale, with the vertical line indicating OR = 1. Haplotypes are ordered by increasing OR. Within-model and study-wide BH-adjusted *p* values, simulated score *p* values, and max-statistic *p* values with corresponding BH-adjusted *p* values are shown; values <0.05 are highlighted in bold. Empty boxes denote loci not included in the respective haplotype combination.

### HLA class I supertype analysis

In the exploratory age- and sex-adjusted supertype analysis (Supplementary Table S5), the A01–A24 cross-reactivity pattern was the only supertype assignment showing a nominal association with AE (OR 1.84, 95% CI 1.11–3.01; raw *p* = 0.019). After BH-FDR correction across the 12 supertype assignments, this association was no longer significant (adjusted *p* = 0.233), and no association remained significant. Multicollinearity was low (VIF 1.16–2.08). In this cohort, the A01–A24 cross-reactivity pattern comprised A*29:01, A*29:02, and A*29:03 and corresponded to the A*29 carrier group. Thus, the nominal association was consistent with the A*29-related signal observed in the allele and haplotype analyses but did not indicate an independently significant supertype effect after adjustment for multiple testing.

### Early-onset AE: exploratory analysis

The exploratory early-onset subgroup comprised 63 patients diagnosed at ≤35 years. In age- and sex-adjusted regression, A*31 (OR 3.39, 95% CI 1.33–7.74), DRB1*14 (OR 2.95, 95% CI 1.10–7.11), and B*07 (OR 0.48, 95% CI 0.22–0.94) showed nominal one-field associations that remained significant after BH correction within the selected model but not across all 78 one-field variables. At two-field resolution, A*31:01 (OR 2.86, 95% CI 1.10–6.70) and DQB1*05:03 (OR 3.44, 95% CI 1.31–8.17) were nominally associated but did not survive within-model or full-family correction. Although DQB1*03:02 and DRB1*04:01 showed nominal associations in the univariate analyses, neither remained independently associated when entered jointly into the multivariable model; each became significant when the other was omitted, suggesting an overlapping LD-related signal. Leave-one-case-out analyses indicated greater robustness for DQB1*05:03 than for A*31:01. Details, including absolute carrier numbers and frequencies, are provided in Supplementary Tables S6a and S6b.

## Discussion

### Key findings and interpretation

At the single-marker level, C*07:01 showed the strongest nominal risk association against overall controls. DRB1*01:01 was the only marker with nominal protective associations in the univariate comparisons against overall, population, and household controls. A*29:02 and C*16 showed nominal risk associations against population controls and risk estimates in the same direction against household controls that narrowly missed nominal significance. However, no association survived BH-FDR correction across the complete univariate screening families.

In the selected multivariable models, the signals for B*08, B*38, and DRB1*14 remained nominal, whereas C*07:01 was associated with approximately doubled odds of AE and B*18:01 with approximately halved odds after within-model correction. However, the correlated B*08:01/C*07:01 pattern precludes attribution of an independent causal effect to either allele.

In the haplotype analyses A*29–DQB1*02 was the most robust pairwise association, while C*16–B*44–DQB1*02 was the only three-locus haplotype surviving within-model regression-based correction. The reference-independent maximum-score analyses consistently supported an extended risk background involving A*29, C*16, or both, in varying combinations with B*44, DRB1*07, and DQB1*02. The broader score-based than regression-based evidence may partly reflect attenuation caused by AE-enriched reference haplotypes. DRB1*07 also showed context-dependent effects, occurring in risk-associated combinations with A*29 or C*16 but in nominally protective combinations with B*57. Overall, these findings support an LD-defined risk background. Since A*29 did not remain significant in the multivariable model, we cannot determine whether it contributes directly to AE susceptibility or reflects linked effects involving C*16, DRB1*07, or other variants.

The exploratory supertype and early-onset analyses did not yield associations surviving correction across their complete testing families. The nominal A01–A24 cross-reactivity signal corresponded to A*29 carriage, consistent with the haplotype analysis. Early-onset signals, including A*31:01 and DQB1*05:03, remain exploratory, although DQB1*05:03 was more robust in sensitivity analyses. HLA homozygosity was not associated with AE.

Taken together, the observed associations may reflect HLA-dependent differences in the presentation of *E. multilocularis*-derived peptides, which could influence the effectiveness of T-cell responses and contribute to interindividual variation in AE susceptibility.

### Integration in international literature

Previous studies of HLA-related susceptibility to AE have produced heterogeneous and partly conflicting results. Comparability is limited by differences in population background, HLA resolution and loci examined, and particularly by the small sample sizes of most previous studies. Accordingly, the overlaps with our findings mostly concern allele groups, subgroups, or linked haplotype backgrounds rather than exact replication of independent allele effects.

Aydinli et al. (2007) [11] reported HLA-A*26 more frequently in 44 Turkish patients with AE. In our cohort, A*26 was also more frequent in AE patients than in overall controls (8.3% versus 6.0%), but this difference did not reach nominal significance in the univariate analysis. DRB1*15 and HLA-DQB1 markers *02, *06, and *07 were more frequent in the Turkish control group; none of the corresponding markers in our cohort showed a significant association with AE versus overall controls. Aydinli et al. [11] also reported DRB1*11 at a lower frequency in patients than in controls, although the reported effect estimate was not statistically significant. Consistently, Eiermann et al. (1998), [10] reported DRB1*11 in 17.2% of 151 European AE patients compared with 27.4% of 453 controls, although this association did not remain significant after correction for multiple testing. In our study, DRB1*11 showed a similar protective direction versus population controls (17.2% versus 22.1%; raw *p* = 0.043), but not versus household controls (19.0%; raw *p* = 0.680). By contrast, Laivacuma et al. [12] reported DRB1*11-containing haplotypes as risk-associated in the Latvian AE group, although this group comprised only eight patients.

DRB1*07 showed a strongly context-dependent pattern. In our haplotype analyses, it occurred in risk-associated backgrounds containing A*29 or C*16, whereas combinations with B*57 showed nominally protective estimates. Previous findings were similarly inconsistent. Li et al. [13] found DRB1*07:01 less frequently in 35 Chinese AE patients than in 104 controls (2.9% versus 13.9%), whereas Laivacuma et al. [12] reported a risk-direction estimate based on 2 of 16 allele copies in 8 Latvian AE patients compared with 4 of 200 control allele copies in 100 controls (OR 7.00, 95% CI 0.57–52.99). Neither study included the class I loci required to determine whether DRB1*07 occurred within the A*29/C*16-associated or B*57-associated backgrounds identified here. These contrasting results therefore support a population- and haplotype-dependent interpretation rather than a uniform effect of DRB1*07.

Li et al. [13] also reported DRB1*040x as a susceptibility marker. In our cohort, DRB1*04:01 was slightly more frequent in AE patients than in controls (15.8% versus 12.3%; raw *p* = 0.070), with a more pronounced difference in the early-onset subgroup (23.8% versus 12.3%). DRB1*04:01 also became nominally significant in a conditional model regarding early-onset AE after removal of the linked DQB1*03:02 predictor, and the C*04–DRB1*04–DQB1*03 haplotype showed a nominal risk association. This observation is noteworthy because contemporaneous epidemiological data indicated a younger mean age at AE diagnosis in central China [23] than in Europe [24] (40 versus 53 years, respectively). Nevertheless, these findings represent a subgroup- and haplotype-level parallel rather than replication of a general independent DRB1*04 effect.

Laivacuma et al. [12] reported a higher DQB1*05:01 allele frequency in their eight AE patients than in controls (5/16 allele copies [31%] versus 25/200 [12%]; OR 3.18, 95% CI 0.79–10.92). In our cohort, the DRB1*14–DQB1*05 haplotype likewise showed a nominal risk association (OR 1.70, 95% CI 1.02–2.86, raw *p* = 0.043). We observed a related signal for DQB1*05:03 in the early-onset subgroup, in which it occurred in 7 of 63 patients (11.1% versus 4.6% in controls; adjusted OR 3.44, 95% CI 1.31–8.17; raw *p* = 0.014). This association remained nominally significant in all leave-one-case-out analyses. Because the two-field alleles differ, these findings indicate an allele-group-level similarity rather than direct replication.

The serological HLA-A1 signal reported in an early Russian study [14] showed only limited overlap with our findings: A*01 was not individually associated with AE, although it occurred in the nominally associated A*01–DQB1*02 haplotype and in frequent haplotype backgrounds involving B*08, C*07, and DRB1*03. Other previously reported allele-level markers, including HLA-A11 and HLA-B35 in Russia [15] and DRB1*09:01 and DRB1*16:01/02 in Alaska [16] were not supported in the overall-control comparison.

Conversely, the recurring A*29/C*16-associated haplotype background identified in our analyses has not been highlighted in previous studies. Notably, none of the previous AE susceptibility studies performed formal multilocus haplotype analyses. The coherent pattern therefore became apparent only when alleles were evaluated jointly within their inferred haplotypic context and may represent an extended LD-defined susceptibility background rather than independent effects of its individual components. Population-specific LD patterns and the limited sample size and locus coverage of earlier cohorts may additionally explain why this background was not previously identified.

### Conclusion and implications

In the multivariable models, C*07:01 and B*18:01 showed the strongest corrected risk and protective signals, respectively, although the C*07:01 signal occurred within a correlated LD background involving B*08:01.

Haplotype analyses identified an extended A*29/C*16-associated background, with A*29–DQB1*02 remaining significant after study-wide correction. The A01–A24 supertype pattern also reflected A*29 carriage. Context-dependent DRB1*07 associations may help explain the contrasting findings reported by Li et al. [13] and Laivacuma et al., [12] highlighting the importance of considering haplotypic background.

The protective tendency for DRB1*11 reported by Eiermann et al. [10] and Aydinli et al. [11] was observed against population but not household controls, illustrating the value of accounting for environmental exposure.

Exploratory early-onset analyses suggested associations with A*31:01 and DQB1*05:03, while the DRB1*04:01 signal provided a potential parallel to findings reported by Li et al. [13]

These associations do not support individual risk prediction or HLA-based screening. Rather, AE susceptibility likely reflects an interplay between genetic background, environmental exposure, and immune status. Future studies should prioritize replication in independent cohorts, exposure-comparable controls, LD-aware haplotype analyses, and early-onset disease.

### Strengths and limitations

To our knowledge, this is the largest cohort assessing HLA associations in AE, exceeding the combined AE patient sample size of previously published studies. Strengths include high-resolution typing across five HLA loci, matching on age, sex, and place of residence, with additional adjustment for age and sex, correction for multiple testing, and complementary haplotype-based approaches accounting for LD. The inclusion of unrelated household controls provides an additional perspective on shared environmental exposure.

However, the limited number of household controls reduced statistical power, and differences between household- and population-control comparisons may also reflect random sampling variation or differences in population composition. Although known biological relatives were excluded, distant or unrecognized relatedness among participants was not formally assessed.

Population controls were younger than AE patients, although the median age difference was small and regression models were adjusted for age. Nevertheless, residual confounding due to unmeasured cohort effects or age-related differences in immune function cannot be excluded and could bias association estimates.

Supertype assignments were based on previously published peptide-binding classifications; no experiments were performed to assess the binding of *E. multilocularis*-derived peptides to specific HLA molecules. Accordingly, the observed associations should be regarded as candidates for functional investigation rather than evidence of allele-specific antigen presentation.

Finally, the single-center design limits generalizability, highlighting the need for international multicenter studies.

## Supplementary Material

SupplementaryMaterial_revised_final.docx

## Data sharing statement

Aggregate data (e.g. selected allele frequencies) are available from the corresponding author upon reasonable request. Individual-level HLA genotype data and linked clinical/demographic information are not publicly available because they constitute human genetic data with potential re-identification risk and are subject to ethical/data-protection restrictions.
