# Supplementary material for: HLA profiles and susceptibility to Alveolar echinococcosis: evidence from a large cohort with population and household controls
Source: Emerg Microbes Infect. 2026 Oct 15;15(1):2744492. doi: 10.1080/22221751.2026.2744492 (PMC13674669; doi:10.1080/22221751.2026.2744492)
Supplement: Supplementary file 1 [file TEMI_A_2744492_SM7285.docx]

**Supplementary Material**

**Table S1. Univariate HLA associations across all control comparisons**

| **HLA marker** | **AE patients n/N (%)** | **Overall controls** | | | | **Population controls** | | | | **Household controls** | | | |
| --- | --- | --- | --- | --- | --- | --- | --- | --- | --- | --- | --- | --- | --- |
|  |  | **n/N (%)** | **OR (95% CI)** | ***p* raw** | ***p* FDR (BH)** | **n/N (%)** | **OR (95% CI)** | ***p* raw** | ***p* FDR (BH)** | **n/N (%)** | **OR (95% CI)** | ***p* raw** | ***p* FDR (BH)** |
| A*02 | 166/360 (46.1%) | 774/1540 (50.3%) | 0.847 (0.673–1.066) | 0.156 | 0.675 | 717/1440 (49.8%) | 0.863 (0.684–1.088) | 0.212 | 0.682 | 57/100 (57.0%) | 0.646 (0.413–1.009) | 0.054 | 0.761 |
| A*02:01 | 156/360 (43.3%) | 740/1540 (48.1%) | 0.827 (0.656–1.042) | 0.106 | 0.870 | 685/1440 (47.6%) | 0.843 (0.668–1.064) | 0.150 | 1.000 | 55/100 (55.0%) | 0.626 (0.401–0.977) | **0.038** | 1.000 |
| A*02:17 | 0/360 (0.0%) | 5/1540 (0.3%) | 0.000 (0.000–4.673) | 0.699 | 1.000 | 2/1440 (0.1%) | 0.000 (0.000–21.315) | 1.000 | 1.000 | 3/100 (3.0%) | 0.000 (0.000–0.665) | **0.020** | 0.757 |
| A*29 | 30/360 (8.3%) | 74/1540 (4.8%) | 1.801 (1.159–2.798) | **0.008** | 0.211 | 70/1440 (4.9%) | 1.779 (1.141–2.774) | **0.010** | 0.237 | 4/100 (4.0%) | 2.182 (0.750–6.347) | 0.143 | 0.930 |
| A*29:02 | 30/360 (8.3%) | 65/1540 (4.2%) | 2.063 (1.317–3.231) | **0.001** | 0.132 | 62/1440 (4.3%) | 2.021 (1.286–3.176) | **0.002** | 0.199 | 3/100 (3.0%) | 2.939 (0.878–9.840) | 0.067 | 1.000 |
| A*66 | 0/360 (0.0%) | 18/1540 (1.2%) | 0.00 (0.00–0.97) | 0.034 | 0.439 | 16/1440 (1.1%) | 0.00 (0.00–1.03) | 0.054 | 0.513 | 2/100 (2.0%) | 0.00 (0.00–1.47) | 0.047 | 0.562 |
| A*66:01 | 0/360 (0.0%) | 18/1540 (1.2%) | 0.00 (0.00–0.97) | 0.034 | 0.770 | 16/1440 (1.1%) | 0.00 (0.00–1.03) | 0.054 | 0.847 | 2/100 (2.0%) | 0.00 (0.00–1.47) | 0.047 | 0.787 |
| B*08 | 89/360 (24.7%) | 289/1540 (18.8%) | 1.422 (1.084–1.865) | **0.011** | 0.211 | 263/1440 (18.3%) | 1.470 (1.117–1.934) | **0.006** | 0.237 | 26/100 (26.0%) | 0.935 (0.563–1.552) | 0.794 | 1.000 |
| B*08:01 | 88/360 (24.4%) | 289/1540 (18.8%) | 1.400 (1.067–1.839) | **0.015** | 0.625 | 263/1440 (18.3%) | 1.448 (1.100–1.906) | **0.008** | 0.422 | 26/100 (26.0%) | 0.921 (0.554–1.529) | 0.750 | 1.000 |
| B*18 | 25/360 (6.9%) | 160/1540 (10.4%) | 0.644 (0.415–0.998) | **0.047** | 0.439 | 150/1440 (10.4%) | 0.642 (0.413–0.997) | **0.047** | 0.513 | 10/100 (10.0%) | 0.672 (0.311–1.450) | 0.308 | 1.000 |
| B*18:01 | 24/360 (6.7%) | 158/1540 (10.3%) | 0.625 (0.400–0.976) | **0.037** | 0.856 | 148/1440 (10.3%) | 0.624 (0.399–0.976) | **0.037** | 0.858 | 10/100 (10.0%) | 0.643 (0.297–1.393) | 0.260 | 1.000 |
| B*35 | 59/360 (16.4%) | 275/1540 (17.9%) | 0.902 (0.663–1.227) | 0.510 | 0.927 | 249/1440 (17.3%) | 0.938 (0.687–1.279) | 0.684 | 0.974 | 26/100 (26.0%) | 0.558 (0.329–0.945) | **0.028** | 0.693 |
| B*35:01 | 32/360 (8.9%) | 149/1540 (9.7%) | 0.911 (0.610–1.359) | 0.647 | 1.000 | 132/1440 (9.2%) | 0.967 (0.645–1.449) | 0.870 | 1.000 | 17/100 (17.0%) | 0.476 (0.252–0.899) | **0.020** | 0.757 |
| B*38 | 24/360 (6.7%) | 63/1540 (4.1%) | 1.675 (1.031–2.719) | **0.035** | 0.439 | 56/1440 (3.9%) | 1.765 (1.078–2.890) | **0.022** | 0.341 | 7/100 (7.0%) | 0.949 (0.397–2.271) | 0.906 | 1.000 |
| B*38:01 | 24/360 (6.7%) | 63/1540 (4.1%) | 1.675 (1.031–2.719) | **0.035** | 0.856 | 56/1440 (3.9%) | 1.765 (1.078–2.890) | **0.022** | 0.768 | 7/100 (7.0%) | 0.949 (0.397–2.271) | 0.906 | 1.000 |
| C*04 | 65/360 (18.1%) | 335/1540 (21.8%) | 0.793 (0.590–1.064) | 0.121 | 0.557 | 305/1440 (21.2%) | 0.820 (0.609–1.103) | 0.189 | 0.682 | 30/100 (30.0%) | 0.514 (0.310–0.852) | **0.009** | 0.660 |
| C*04:01 | 65/360 (18.1%) | 335/1540 (21.8%) | 0.793 (0.590–1.064) | 0.121 | 0.870 | 305/1440 (21.2%) | 0.820 (0.609–1.103) | 0.189 | 1.000 | 30/100 (30.0%) | 0.514 (0.310–0.852) | **0.009** | 0.757 |
| C*07 | 199/360 (55.3%) | 775/1540 (50.3%) | 1.220 (0.969–1.536) | 0.091 | 0.557 | 723/1440 (50.2%) | 1.226 (0.972–1.546) | 0.085 | 0.549 | 52/100 (52.0%) | 1.141 (0.732–1.779) | 0.560 | 1.000 |
| C*07:01 | 129/360 (35.8%) | 413/1540 (26.8%) | 1.524 (1.195–1.943) | **<0.001** | 0.132 | 380/1440 (26.4%) | 1.558 (1.219–1.991) | **<0.001** | 0.077 | 33/100 (33.0%) | 1.134 (0.709–1.813) | 0.600 | 1.000 |
| C*16 | 35/360 (9.7%) | 89/1540 (5.8%) | 1.756 (1.166–2.643) | **0.006** | 0.211 | 85/1440 (5.9%) | 1.717 (1.137–2.592) | **0.009** | 0.237 | 4/100 (4.0%) | 2.585 (0.896–7.455) | 0.069 | 0.761 |
| C*16:01 | 29/360 (8.1%) | 65/1540 (4.2%) | 1.988 (1.263–3.129) | **0.003** | 0.175 | 61/1440 (4.2%) | 1.981 (1.253–3.131) | **0.003** | 0.204 | 4/100 (4.0%) | 2.103 (0.721–6.129) | 0.164 | 1.000 |
| DRB1*01 | 47/360 (13.1%) | 267/1540 (17.3%) | 0.716 (0.513–0.9996) | **0.049** | 0.439 | 247/1440 (17.2%) | 0.725 (0.518–1.015) | 0.060 | 0.513 | 20/100 (20.0%) | 0.601 (0.337–1.071) | 0.082 | 0.761 |
| DRB1*01:01 | 36/360 (10.0%) | 224/1540 (14.5%) | 0.653 (0.450–0.947) | **0.024** | 0.762 | 205/1440 (14.2%) | 0.669 (0.460–0.974) | **0.035** | 0.858 | 19/100 (19.0%) | 0.474 (0.258–0.869) | **0.014** | 0.757 |
| DRB1*11 | 62/360 (17.2%) | 337/1540 (21.9%) | 0.743 (0.551–1.002) | 0.051 | 0.439 | 318/1440 (22.1%) | 0.734 (0.543–0.991) | **0.043** | 0.513 | 19/100 (19.0%) | 0.887 (0.502–1.568) | 0.680 | 1.000 |
| DRB1*13 | 105/360 (29.2%) | 399/1540 (25.9%) | 1.178 (0.913–1.518) | 0.208 | 0.675 | 380/1440 (26.4%) | 1.149 (0.889–1.483) | 0.288 | 0.739 | 19/100 (19.0%) | 1.755 (1.014–3.039) | **0.043** | 0.761 |
| DRB1*13:01 | 57/360 (15.8%) | 222/1540 (14.4%) | 1.117 (0.814–1.533) | 0.494 | 1.000 | 214/1440 (14.9%) | 1.078 (0.784–1.481) | 0.645 | 1.000 | 8/100 (8.0%) | 2.163 (0.996–4.700) | **0.047** | 1.000 |
| DRB1*14 | 28/360 (7.8%) | 69/1540 (4.5%) | 1.798 (1.141–2.834) | **0.010** | 0.211 | 65/1440 (4.5%) | 1.784 (1.127–2.823) | **0.012** | 0.237 | 4/100 (4.0%) | 2.024 (0.693–5.913) | 0.189 | 1.000 |
| DRB1*14:01 | 16/360 (4.4%) | 31/1540 (2.0%) | 2.264 (1.225–4.186) | **0.007** | 0.390 | 30/1440 (2.1%) | 2.186 (1.178–4.056) | **0.011** | 0.462 | 1/100 (1.0%) | 4.595 (0.697–195.018) | 0.169 | 1.000 |
| DQB1*05 | 99/360 (27.5%) | 428/1540 (27.8%) | 0.985 (0.762–1.274) | 0.911 | 1.000 | 400/1440 (27.8%) | 0.986 (0.762–1.277) | 0.916 | 1.000 | 28/100 (28.0%) | 0.975 (0.595–1.599) | 0.921 | 1.000 |
| DQB1*05:01 | 51/360 (14.2%) | 287/1540 (18.6%) | 0.721 (0.522–0.995) | **0.046** | 0.868 | 267/1440 (18.5%) | 0.725 (0.524–1.003) | 0.052 | 0.888 | 20/100 (20.0%) | 0.660 (0.372–1.170) | 0.153 | 1.000 |
| DQB1*05:03 | 27/360 (7.5%) | 71/1540 (4.6%) | 1.678 (1.060–2.654) | **0.026** | 0.762 | 67/1440 (4.7%) | 1.662 (1.046–2.639) | **0.030** | 0.858 | 4/100 (4.0%) | 1.946 (0.665–5.698) | 0.217 | 1.000 |
| DQB1*06 | 178/360 (49.4%) | 710/1540 (46.1%) | 1.143 (0.909–1.438) | 0.253 | 0.680 | 673/1440 (46.7%) | 1.115 (0.885–1.404) | 0.357 | 0.816 | 37/100 (37.0%) | 1.665 (1.056–2.626) | **0.027** | 0.693 |

Markers with central two-sided Fisher or Pearson p raw < 0.05 for AE versus controls are shown including respective allele group. For each control comparison, BH-FDR correction was applied separately to the complete set of one-field and two-field tests. Frequencies are carrier prevalences (n/N), ORs are unadjusted odds ratios with 95% confidence intervals.

Abbreviations: AE, alveolar echinococcosis; FDR, false discovery rate; BH, Benjamini-Hochberg; CI, confidence interval; OR, odds ratio.

**Tables S2. Multivariable HLA models**

**Table S2a. AE versus population controls: one-field allele-group model**

| **Allele group** | **OR** | **95% CI** | **p raw** | **p FDR (BH)** | **VIF** |
| --- | --- | --- | --- | --- | --- |
| A*29 | 1.34 | 0.75-2.34 | 0.322 | 0.322 | 1.46 |
| **B*08** | 1.52 | 1.14-2.01 | **0.005** | **0.038** | 1.02 |
| B*18 | 0.70 | 0.44-1.08 | 0.109 | 0.145 | 1.02 |
| **B*38** | 1.77 | 1.05-2.91 | **0.033** | 0.077 | 1.01 |
| C*16 | 1.57 | 0.91-2.65 | 0.101 | 0.145 | 1.47 |
| DRB1*01 | 0.81 | 0.57-1.14 | 0.232 | 0.265 | 1.03 |
| **DRB1*14** | 1.78 | 1.09-2.83 | **0.021** | 0.077 | 1.01 |

Logistic regression was adjusted for age (per 10-year increase) and sex.

Abbreviations: FDR, false discovery rate; BH, Benjamini-Hochberg; CI, confidence interval; OR, odds ratio; VIF, variance inflation factor.

**Table S2b. AE versus population controls: two-field allele model**

| **Allele** | **OR** | **95% CI** | **p raw** | **p FDR (BH)** | **VIF** |
| --- | --- | --- | --- | --- | --- |
| A*29:02 | 1.51 | 0.77-2.89 | 0.223 | 0.409 | 1.86 |
| B*08:01 | 0.78 | 0.49-1.24 | 0.289 | 0.409 | 2.58 |
| **B*18:01** | 0.49 | 0.29-0.79 | **0.003** | **0.018** | 1.17 |
| **B*38:01** | 1.78 | 1.05-2.93 | **0.033** | 0.096 | 1.01 |
| **C*07:01** | 2.10 | 1.38-3.18 | **<0.001** | **0.007** | 2.65 |
| C*16:01 | 1.58 | 0.80-3.05 | 0.181 | 0.398 | 1.86 |
| DQB1*05:01 | 0.98 | 0.54-1.72 | 0.959 | 0.959 | 3.52 |
| DQB1*05:03 | 1.33 | 0.67-2.51 | 0.403 | 0.493 | 1.78 |
| DRB1*01:01 | 0.77 | 0.40-1.53 | 0.448 | 0.493 | 3.51 |
| DRB1*14:01 | 1.59 | 0.66-3.85 | 0.297 | 0.409 | 1.77 |

Logistic regression was adjusted for age (per 10-year increase) and sex.

Abbreviations: FDR, false discovery rate; BH, Benjamini-Hochberg; CI, confidence interval; OR, odds ratio; VIF, variance inflation factor.

**Conditional B*08:01/C*07:01 analysis:** After omission of C*07:01, the estimate for B*08:01 was OR 1.49 (95% CI 1.12-1.98; p = 0.007). After omission of B*08:01, C*07:01 remained associated with higher AE odds (OR 1.76, 95% CI 1.35-2.27; p < 0.001). The change in the B*08:01 estimate indicates substantial dependence on the linked C*07:01 signal.

**Table S2c. AE versus household controls: one-field allele-group fixed-predictor model**

| **Allele group** | **OR** | **95% CI** | **p raw** | **p FDR (BH)** | **VIF** |
| --- | --- | --- | --- | --- | --- |
| A*29 | 1.34 | 0.38-5.48 | 0.660 | 0.791 | 1.79 |
| B*08 | 0.89 | 0.53-1.53 | 0.678 | 0.791 | 1.08 |
| B*18 | 0.64 | 0.30-1.44 | 0.270 | 0.602 | 1.02 |
| B*38 | 0.91 | 0.40-2.30 | 0.832 | 0.832 | 1.03 |
| C*16 | 1.82 | 0.55-7.54 | 0.344 | 0.602 | 1.80 |
| DRB1*01 | 0.63 | 0.36-1.15 | 0.128 | 0.602 | 1.04 |
| DRB1*14 | 1.73 | 0.68-5.53 | 0.265 | 0.602 | 1.01 |

Logistic regression was adjusted for age (per 10-year increase) and sex.

Abbreviations: FDR, false discovery rate; BH, Benjamini-Hochberg; CI, confidence interval; OR, odds ratio; VIF, variance inflation factor.

**Table S2d. AE versus household controls: two-field allele fixed-predictor model**

| **Allele** | **OR** | **95% CI** | **p raw** | **p FDR (BH)** | **VIF** |
| --- | --- | --- | --- | --- | --- |
| A*29:02 | 3.11 | 0.67-18.15 | 0.156 | 0.312 | 2.23 |
| B*08:01 | 0.44 | 0.17-1.09 | 0.077 | 0.193 | 2.82 |
| B*18:01 | 0.42 | 0.17-1.02 | 0.056 | 0.186 | 1.23 |
| B*38:01 | 0.94 | 0.40-2.42 | 0.888 | 0.916 | 1.04 |
| C*07:01 | 2.23 | 0.99-5.58 | 0.055 | 0.186 | 2.86 |
| C*16:01 | 0.79 | 0.19-3.93 | 0.761 | 0.916 | 2.24 |
| DQB1*05:01 | 1.88 | 0.54-10.03 | 0.349 | 0.567 | 3.61 |
| DQB1*05:03 | 1.07 | 0.35-4.19 | 0.916 | 0.916 | 1.92 |
| DRB1*01:01 | 0.27 | 0.05-1.02 | 0.054 | 0.186 | 3.59 |
| DRB1*14:01 | 2.32 | 0.35-26.13 | 0.397 | 0.567 | 1.93 |

Logistic regression was adjusted for age (per 10-year increase) and sex.

Abbreviations: FDR, false discovery rate; BH, Benjamini-Hochberg; CI, confidence interval; OR, odds ratio; VIF, variance inflation factor.

**Conditional B*08:01/C*07:01 analysis:** After omission of C*07:01, B*08:01 was not associated with AE (OR 0.89, 95% CI 0.53-1.53; p = 0.671). After omission of B*08:01, C*07:01 was also not associated with AE (OR 1.25, 95% CI 0.77-2.06; p = 0.371).

**Table S2e. AE versus household controls: one-field allele-group household-specific model**

| **Allele group** | **OR** | **95% CI** | **p raw** | **p FDR (BH)** | **VIF** |
| --- | --- | --- | --- | --- | --- |
| B*35 | 0.82 | 0.34-2.00 | 0.669 | 0.671 | 2.59 |
| C*04 | 0.61 | 0.27-1.46 | 0.265 | 0.441 | 2.64 |
| **C*17** | 0.15 | 0.03-0.69 | **0.015** | 0.076 | 1.06 |
| DRB1*13 | 1.66 | 0.87-3.25 | 0.125 | 0.313 | 1.44 |
| DQB1*06 | 1.13 | 0.66-1.96 | 0.671 | 0.671 | 1.48 |

Logistic regression was adjusted for age (per 10-year increase) and sex.

Abbreviations: FDR, false discovery rate; BH, Benjamini-Hochberg; CI, confidence interval; OR, odds ratio; VIF, variance inflation factor.

**Table S2f. AE versus household controls: two-field allele household-specific model**

| **Allele** | **OR** | **95% CI** | **p raw** | **p FDR (BH)** | **VIF** |
| --- | --- | --- | --- | --- | --- |
| **A*02:01** | 0.55 | 0.35-0.88 | **0.012** | 0.073 | 1.04 |
| B*35:01 | 0.82 | 0.35-1.91 | 0.638 | 0.638 | 1.67 |
| C*04:01 | 0.57 | 0.30-1.12 | 0.100 | 0.119 | 1.61 |
| C*17:01 | 0.23 | 0.05-1.02 | 0.052 | 0.119 | 1.02 |
| DRB1*01:01 | 0.54 | 0.29-1.06 | 0.073 | 0.119 | 1.12 |
| DRB1*13:01 | 1.91 | 0.93-4.41 | 0.080 | 0.119 | 1.03 |

Logistic regression was adjusted for age (per 10-year increase) and sex.

Abbreviations: FDR, false discovery rate; BH, Benjamini-Hochberg; CI, confidence interval; OR, odds ratio; VIF, variance inflation factor.

**Table S3a. Multilocus homozygosity score**

| **Comparison** | **Mean homozygous loci, AE** | **Mean homozygous loci, controls** | **Adjusted OR per additional homozygous locus (95% CI)** | **Raw regression p** | **BH-FDR p** |
| --- | --- | --- | --- | --- | --- |
| AE vs all controls | 0.44 | 0.47 | 0.95 (0.83–1.09) | 0.476 | — |
| AE vs population controls | 0.44 | 0.46 | 0.96 (0.84–1.10) | 0.560 | — |
| AE vs household controls | 0.44 | 0.55 | 0.89 (0.70–1.12) | 0.319 | — |

**Sensitivity analysis**: Defining exposure as at least one homozygous locus likewise showed no association with AE: 103/360 (28.61%) versus 458/1,540 (29.74%; adjusted OR 0.94, 95% CI 0.72–1.21; raw p = 0.623).

Abbreviations: FDR, false discovery rate; BH, Benjamini-Hochberg; CI, confidence interval; OR, odds ratio;

**Table S3b. Locus-specific homozygosity analyses**

| **Locus** | **AE homozygous, %** | **Controls homozygous, %** | **Adjusted OR: homozygous vs heterozygous (95% CI)** | **Raw regression p** | **BH-FDR p** |
| --- | --- | --- | --- | --- | --- |
| HLA-A | 13.61% | 13.38% | 0.99 (0.71–1.40) | 0.976 | 0.994 |
| HLA-B | 5.28% | 5.13% | 1.00 (0.59–1.68) | 0.994 | 0.994 |
| HLA-C | 7.22% | 8.98% | 0.81 (0.52–1.26) | 0.342 | 0.856 |
| HLA-DRB1 | 8.61% | 8.18% | 1.04 (0.69–1.58) | 0.853 | 0.994 |
| HLA-DQB1 | 9.17% | 11.44% | 0.76 (0.51–1.13) | 0.180 | 0.856 |

The multilocus homozygosity score counted homozygous loci across HLA-A, HLA-B, HLA-C, HLA-DRB1, and HLA-DQB1 (range, 0–5) and was analysed using logistic regression adjusted for age (per 10-year increase) and sex.

Abbreviations: FDR, false discovery rate; BH, Benjamini-Hochberg; CI, confidence interval; OR, odds ratio;

**Table S4a. Pairwise haplotype analysis.**

| **Haplotype** | **AE, %** | **Controls, %** | **Adjusted OR (95% CI)** | **Raw regression p** | **Within-model BH p** | **Study-wide* BH p** | **Simulated score p** | **Max-stat p / BH p** |
| --- | --- | --- | --- | --- | --- | --- | --- | --- |
| **A–B \| Reference: A*01–B*08** | | | | | | | | |
| **A*02–B*44** | 2.69 | 4.04 | 0.49 (0.27–0.89) | **0.020** | 0.214 | 0.421 | 0.0975 | — |
| **A*02–B*27** | 0.65 | 1.72 | 0.24 (0.06–0.87) | **0.030** | 0.214 | 0.448 | **0.0378** | — |
| **A*02–B*40** | 2.57 | 3.84 | 0.50 (0.27–0.94) | **0.031** | 0.214 | 0.448 | 0.1310 | — |
| **A*03–B*35** | 1.86 | 2.80 | 0.42 (0.19–0.95) | **0.037** | 0.214 | 0.454 | 0.1230 | — |
| **A*29–B*44** | 3.29 | 1.32 | 1.79 (0.96–3.34) | 0.067 | 0.221 | 0.471 | **0.0008** | **0.0227 / 0.0454** |
| **A–C \| Reference: A*01–C*07** | | | | | | | | |
| **A*29–C*16** | 3.32 | 1.37 | 1.98 (1.09–3.59) | **0.025** | 0.491 | 0.421 | **0.0011** | **0.0202 / 0.0454** |
| **A*03–C*04** | 1.32 | 2.71 | 0.43 (0.19–0.99) | **0.047** | 0.491 | 0.454 | 0.0933 | — |
| **A–DRB1 \| Reference: A*01–DRB1*03** | | | | | | | | |
| **A*29–DRB1*07** | 2.91 | 0.91 | 2.31 (1.12–4.74) | **0.023** | 0.360 | 0.421 | **0.0004** | **0.0129 / 0.0454** |
| **A*03–DRB1*01** | 0.81 | 2.46 | 0.30 (0.10–0.92) | **0.036** | 0.360 | 0.454 | **0.0359** | — |
| **A*24–DRB1*13** | 2.94 | 0.71 | 2.49 (1.01–6.12) | **0.047** | 0.360 | 0.454 | **0.0032** | — |
| **A–DQB1 \| Reference: A*02–DQB1*03** | | | | | | | | |
| **A*29–DQB1*02** | 3.13 | 1.00 | 3.92 (1.93–7.98) | **<0.001** | **0.0050** | **0.0385** | **0.0002** | **0.0153 / 0.0454** |
| **A*01–DQB1*02** | 8.88 | 6.88 | 1.80 (1.16–2.80) | **0.009** | 0.129 | 0.421 | 0.1074 | — |
| **A*24–DQB1*03** | 4.93 | 3.62 | 1.96 (1.12–3.41) | **0.018** | 0.179 | 0.421 | 0.1593 | — |
| **A*11–DQB1*03** | 2.66 | 1.30 | 2.26 (1.04–4.93) | **0.041** | 0.275 | 0.454 | 0.2023 | — |
| **A*24–DQB1*06** | 2.76 | 2.14 | 2.04 (1.01–4.09) | **0.046** | 0.275 | 0.454 | 0.3007 | — |
| **B–C \| Reference: B*07–C*07** | | | | | | | | |
| **B*44–C*16** | 3.75 | 2.30 | 2.17 (1.27–3.71) | **0.004** | 0.115 | 0.341 | **0.0122** | — |
| **B*08–C*07** | 12.92 | 9.86 | 1.56 (1.10–2.22) | **0.013** | 0.166 | 0.421 | **0.0125** | — |
| **B*49–C*07** | 2.22 | 1.29 | 2.13 (1.11–4.09) | **0.023** | 0.166 | 0.421 | **0.0457** | — |
| **B*38–C*12** | 3.33 | 2.05 | 1.90 (1.08–3.33) | **0.026** | 0.166 | 0.421 | 0.0532 | — |
| **B–DRB1 \| Reference: B*08–DRB1*03** | | | | | | | | |
| **B*57–DRB1*07** | 1.66 | 2.79 | 0.39 (0.18–0.81) | **0.012** | 0.228 | 0.421 | **0.0399** | — |
| **B*15–DRB1*13** | 3.86 | 1.99 | 1.57 (0.88–2.79) | 0.126 | 0.300 | 0.551 | **0.0017** | **0.0308** / 0.0513 |
| **B–DQB1 \| Reference: B*07–DQB1*06** | | | | | | | | |
| **B*44–DQB1*02** | 5.19 | 3.04 | 1.70 (1.01–2.88) | **0.047** | 0.635 | 0.454 | **0.0273** | — |
| **C–DRB1 \| Reference: C*07–DRB1*15** | | | | | | | | |
| **C*16–DRB1*07** | 3.44 | 1.48 | 2.46 (1.35–4.48) | **0.003** | 0.0964 | 0.341 | **0.0003** | **0.0190 / 0.0454** |
| **C*04–DRB1*04** | 3.34 | 1.27 | 2.41 (1.23–4.73) | **0.011** | 0.149 | 0.421 | **0.0031** | — |
| **C–DQB1 \| Reference: C*07–DQB1*06** | | | | | | | | |
| **C*16–DQB1*02** | 3.26 | 1.61 | 2.02 (1.10–3.71) | **0.024** | 0.450 | 0.421 | **0.0022** | 0.0573 / 0.0819 |
| **DRB1–DQB1 \| Reference: DRB1*15–DQB1*06** | | | | | | | | |
| **DRB1*14–DQB1*05** | 3.75 | 2.24 | 1.70 (1.02–2.86) | **0.043** | 0.561 | 0.454 | **0.0229** | 0.2503 / 0.2503 |

*BH p across all pairwise haplotype coefficients

Inclusion criteria: overall haplotype frequency >1% and raw regression p <0.05 or the strongest absolute score haplotype of a locus pair when the max-stat p <0.05

Frequencies are EM-estimated haplotype frequencies. Adjusted ORs and 95% CIs were obtained from additive haplotype-regression models adjusted for age per 10 years and sex and are relative to the pair-specific reference haplotype.

**Table S4b. Three-locus haplotype analysis.**

| **Haplotype** | **AE, %** | **Controls, %** | **Adjusted OR (95% CI)** | **Raw regression p** | **Within-model BH p** | **Study-wide* BH p** | **Simulated score p** | **Max-stat p / BH p** |
| --- | --- | --- | --- | --- | --- | --- | --- | --- |
| **A–B–C \| Reference: A*01–B*08–C*07** | | | | | | | | |
| **A*29–B*44–C*16** | 3.19 | 1.29 | 1.84 (0.95–3.59) | 0.073 | 0.292 | 0.524 | **0.0008** | **0.0159 / 0.0199** |
| **A*03–B*35–C*04** | 0.92 | 2.53 | 0.31 (0.11–0.89) | **0.030** | 0.292 | 0.524 | **0.0441** | — |
| **A*02–B*07–C*07** | 2.96 | 3.74 | 0.52 (0.28–0.97) | **0.040** | 0.292 | 0.524 | 0.2078 | — |
| **A–B–DRB1 \| Reference: A*01–B*08–DRB1*03** | | | | | | | | |
| **A*29–B*44–DRB1*07** | 3.03 | 0.83 | 2.80 (1.15–6.81) | **0.023** | 0.211 | 0.522 | **<0.0001** | **0.0002 / 0.0015** |
| **A–B–DQB1 \| Reference: A*01–B*08–DQB1*02** | | | | | | | | |
| **A*29–B*44–DQB1*02** | 3.16 | 0.82 | 2.60 (1.19–5.69) | **0.017** | 0.288 | 0.522 | **<0.0001** | **0.0007 / 0.0022** |
| **A–C–DRB1 \| Reference: A*01–C*07–DRB1*03** | | | | | | | | |
| **A*29–C*16–DRB1*07** | 2.90 | 0.81 | 2.62 (1.12–6.12) | **0.027** | 0.357 | 0.524 | **<0.0001** | **0.0007 / 0.0022** |
| **A–C–DQB1 \| Reference: A*01–C*07–DQB1*02** | | | | | | | | |
| **A*29–C*16–DQB1*02** | 3.03 | 0.87 | 2.51 (1.17–5.36) | **0.018** | 0.272 | 0.522 | **<0.0001** | **0.0014 / 0.0036** |
| **A–DRB1–DQB1 \| Reference: A*01–DRB1*03–DQB1*02** | | | | | | | | |
| **A*29–DRB1*07–DQB1*02** | 2.94 | 0.93 | 2.29 (1.12–4.65) | **0.023** | 0.409 | 0.522 | **0.0004** | **0.0094 / 0.0157** |
| **A*03–DRB1*01–DQB1*05** | 0.52 | 2.34 | 0.33 (0.11–0.95) | **0.040** | 0.409 | 0.524 | 0.0542 | — |
| **B–C–DRB1 \| Reference: B*08–C*07–DRB1*03** | | | | | | | | |
| **B*44–C*16–DRB1*07** | 3.26 | 1.40 | 1.68 (0.84–3.38) | 0.145 | 0.369 | 0.524 | **0.0023** | **0.0446 / 0.0495** |
| **B*57–C*06–DRB1*07** | 1.66 | 2.65 | 0.44 (0.20–0.94) | **0.035** | 0.234 | 0.524 | 0.0786 | — |
| **B*35–C*04–DRB1*11** | 1.02 | 1.50 | 0.23 (0.05–0.97) | **0.045** | 0.234 | 0.524 | 0.0824 | — |
| **B–C–DQB1 \| Reference: B*07–C*07–DQB1*06** | | | | | | | | |
| **B*44–C*16–DQB1*02** | 3.31 | 1.36 | 2.83 (1.48–5.41) | **0.0016** | **0.041** | 0.247 | **0.0002** | **0.0067 / 0.0133** |
| **B–DRB1–DQB1 \| Reference: B*08–DRB1*03–DQB1*02** | | | | | | | | |
| **B*15–DRB1*13–DQB1*06** | 3.59 | 2.02 | 1.47 (0.81–2.64) | 0.204 | 0.386 | 0.531 | **0.0045** | 0.0964 / 0.0964 |
| **B*57–DRB1*07–DQB1*03** | 1.67 | 2.67 | 0.43 (0.21–0.88) | **0.022** | 0.190 | 0.522 | 0.0770 | — |
| **C–DRB1–DQB1 \| Reference: C*07–DRB1*15–DQB1*06** | | | | | | | | |
| **C*16–DRB1*07–DQB1*02** | 3.36 | 1.39 | 2.60 (1.39–4.85) | **0.0028** | 0.075 | 0.247 | **0.0002** | **0.0144 / 0.0199** |
| **C*04–DRB1*04–DQB1*03** | 3.18 | 1.18 | 2.65 (1.28–5.48) | **0.0087** | 0.117 | 0.514 | **0.0027** | — |

*BH p across all three-locus haplotype coefficients

Inclusion criteria: overall haplotype frequency >1% and raw regression p <0.05 or the strongest absolute score haplotype of a three-locus combination.

Frequencies are EM-estimated haplotype frequencies. Adjusted ORs and 95% CIs were obtained from additive haplotype-regression models adjusted for age per 10 years and sex and are relative to the three-locus-specific reference haplotype.

**Table S5. Multivariate class I supertype analysis.**

| **Supertype** | **OR** | **95% CI** | **p raw** | **p FDR (BH)** | **VIF** |
| --- | --- | --- | --- | --- | --- |
| A01 | 0.98 | 0.70-1.37 | 0.893 | 0.963 | 2.08 |
| A01-A03 | 0.71 | 0.33-1.41 | 0.342 | 0.813 | 1.16 |
| **A01-A24** | 1.84 | 1.11-3.01 | **0.019** | 0.233 | 1.19 |
| A02 | 0.89 | 0.64-1.24 | 0.495 | 0.848 | 2.01 |
| A03 | 1.03 | 0.74-1.44 | 0.865 | 0.963 | 2.07 |
| A24 | 1.01 | 0.71-1.44 | 0.955 | 0.963 | 1.60 |
| B07 | 0.75 | 0.55-1.04 | 0.082 | 0.490 | 1.90 |
| B08 | 1.28 | 0.88-1.84 | 0.192 | 0.576 | 1.68 |
| B27 | 0.99 | 0.71-1.39 | 0.963 | 0.963 | 1.48 |
| B44 | 0.80 | 0.58-1.10 | 0.169 | 0.576 | 1.90 |
| B58 | 0.83 | 0.54-1.28 | 0.407 | 0.813 | 1.27 |
| B62 | 1.09 | 0.75-1.58 | 0.646 | 0.963 | 1.36 |

Logistic regression was adjusted for age (per 10-year increase) and sex.

Abbreviations: FDR, false discovery rate; BH, Benjamini-Hochberg; CI, confidence interval; OR, odds ratio; VIF, variance inflation factor.

**Classification note:** Supertypes were assigned according to Sidney et al. (DOI: [10.1186/1471-2172-9-1](https://doi.org/10.1186/1471-2172-9-1)). A01-A03 and A01-A24 denote dual-specificity cross-reactivity patterns rather than combinations of the conventional supertypes. In this cohort, the A01-A24 category comprised A*29:01, A*29:02, and A*29:03 and corresponded exactly to the A*29 carrier group.

**Table S6a. Multivariate analysis of early-onset AE: One-field allele group model**

| **Allele group** | **n/N (%)** | **OR** | **95% CI** | **p raw** | **p FDR (BH)** | **VIF** |
| --- | --- | --- | --- | --- | --- | --- |
| **A*31** | 7/63 (11.1) | 3.39 | 1.33-7.74 | **0.013** | **0.033** | 1.00 |
| **DRB1*14** | 7/63 (11.1) | 2.95 | 1.10-7.11 | **0.033** | **0.033** | 1.00 |
| **B*07** | 10/63 (15.9) | 0.48 | 0.22-0.94 | **0.030** | **0.033** | 1.00 |

Logistic regression was adjusted for age (per 10-year increase) and sex.

None of the selected terms survived full-family BH correction across 78 testable HLA variables (adjusted p = 0.593).

Abbreviations: FDR, false discovery rate; BH, Benjamini-Hochberg; CI, confidence interval; OR, odds ratio; VIF, variance inflation factor.

**Table S6b. Multivariate analysis of early-onset AE: Two-field allele model**

| **Allele** | **n/N (%)** | **OR** | **95% CI** | **p raw** | **p FDR (BH)** | **VIF** |
| --- | --- | --- | --- | --- | --- | --- |
| DQB1*03:02 | 20/63 (31.7) | 1.63 | 0.77-3.28 | 0.196 | 0.220 | 1.33 |
| **A*31:01** | 7/63 (11.1) | 2.86 | 1.10-6.70 | **0.033** | 0.066 | 1.01 |
| DRB1*04:01 | 15/63 (23.8) | 1.67 | 0.73-3.69 | 0.220 | 0.220 | 1.33 |
| **DQB1*05:03** | 7/63 (11.1) | 3.44 | 1.31-8.17 | **0.014** | 0.057 | 1.00 |

Logistic regression was adjusted for age (per 10-year increase) and sex.

None of the selected terms survived full-family BH correction across 202 testable HLA variables (adjusted p= 0.588).

Abbreviations: FDR, false discovery rate; BH, Benjamini-Hochberg; CI, confidence interval; OR, odds ratio; VIF, variance inflation factor.

**Conditional DQB1*03:02/DRB1*04:01 analysis:** After omission of DRB1*04:01, DQB1*03:02 showed OR 2.06 (95% CI 1.13-3.68; p = 0.020). After omission of DQB1*03:02, DRB1*04:01 showed OR 2.27 (95% CI 1.14-4.30; p = 0.020). The reciprocal change is consistent with an overlapping linked signal, and the independent allelic effects cannot be reliably disentangled in this subgroup.

**Leave-one-case-out sensitivity analysis:** the estimated association for A*31:01 remained consistently positive (OR range 2.41–2.99). However, nominal significance depended on retaining all seven A*31:01-positive early-onset cases and was lost whenever one of these cases was omitted. By contrast, the DQB1*05:03 association was robust to the exclusion of any individual early-onset case (OR range 2.85–3.54), with raw *p*<0.05 and 95% CIs excluding 1 in all 63 refitted models.
